# The Influence of Interfacial Chemistry on Magnesium Electrodeposition in Non-nucleophilic Electrolytes Using Sulfone-Ether Mixtures

**DOI:** 10.3389/fchem.2019.00194

**Published:** 2019-04-03

**Authors:** Laura C. Merrill, Jennifer L. Schaefer

**Affiliations:** Department of Chemical and Biomolecular Engineering, University of Notre Dame, Notre Dame, IN, United States

**Keywords:** magnesium battery, electrodeposition, electrolyte, sulfones, interface, dendrite

## Abstract

One of the limiting factors in the development of magnesium batteries is the reversibility of magnesium electrodeposition and dissolution at the anode. Often irreversibility is related to impurities and decomposition. Herein we report on the cycling behavior of magnesium metal anodes in different electrolytes, Mg(HMDS)_2_ – 4 MgCl_2_ in tetrahydrofuran (THF) and a butyl sulfone/THF mixture. The deposition morphology and anode-electrolyte interface is studied and related to Mg/Mg cell cycling performance. It is found that adding the sulfone caused the formation of a boundary layer at the electrode-electrolyte interface, which, in turn, resulted in a particle-like deposition morphology. This type of deposition has a high surface area, which alters the effective local current density and results in electronically isolated deposits. Extended cycling resulted in magnesium growth through a separator. Electrolyte decomposition is observed with and without the addition of the sulfone, however the addition of the sulfone increased the degree of decomposition.

## Introduction

Demands for high performance batteries have increased with the use of portable electronic devices and electric vehicles, leading to growth in research of post lithium-ion batteries. The use of metallic anodes, as opposed to intercalation anodes, creates an opportunity to meet these high performance metrics. A metallic anode is entirely comprised of active material, thus the theoretical energy density is inherently greater than traditional lithium-ion anodes that use host matrices. Magnesium is a viable anode option as it has large theoretical volumetric and gravimetric capacities (3,800 mAh/cm^3^ and 2,200 mAh/g respectively), relatively low electrodeposition potential (−2.4 V vs. SHE), and is widely abundant in the Earth's crust.

Magnesium has gained attention as a post lithium-ion anode material following the first magnesium battery prototype developed in the early 2000s (Aurbach et al., 2000). This prototype used an electrolyte composed of a Grignard reagent complexed with a Lewis acid to achieve highly reversible electrodeposition (Aurbach et al., 2000; Amir et al., 2007; Viestfrid et al., 2007). Recent progress has focused on non-Grignard chemistries through use of alternative electrolytes, such as magnesium alkoxides or magnesium bis(hexamethyldisilizide) (Mg(HMDS)_2_) complexed with a chloride salt (MgCl_2_ or AlCl_3_) in tetrahydrofuran (THF) (Kim et al., 2011; Herb et al., 2015; Liao et al., 2015; Pan et al., 2015), and Mg(TFSI)_2_, or Mg(BH_4_)_2_ in dimethoxyethane (DME) or higher order glymes (Mohtadi et al., 2012; Ha et al., 2014). Despite this progress, magnesium battery research has been hindered by low electrodeposition/dissolution efficiencies; the use of a metallic anode requires Coulombic efficiencies >99% for practical application (Wang et al., 2018). In the presence of trace impurities and certain salts and solvents, the metal is able to form a stable passivating layer that prevents the magnesium ions from reaching the active magnesium metal surface, thus not allowing electron transfer to occur. Because of this, only certain salt and solvent combinations have resulted in reversible magnesium electrodeposition.

Recently there has been interest in magnesium deposition and how deposition kinetics can influence the resultant morphology (Viestfrid et al., 2005; Matsui, 2011; Wetzel et al., 2015; Crowe et al., 2017). Early magnesium battery research suggested that it is unfavorable for magnesium to form dendrites, unlike lithium metal; this is attributed to differences in the crystal structures of each metal (Ling et al., 2012; Jäckle and Groß, 2014; Lautar et al., 2019). Although magnesium is less likely to form dendrites compared to lithium, dendritic magnesium deposits been observed with both complex and simple salt electrolytes (Ding et al., 2018; Davidson et al., 2019). In particular, Mg(TFSI)_2_ systems were found to short circuit cells due to the formation of an unstable solid electrolyte interphase (SEI), resultant from the partial decomposition of Mg(TFSI)_2_ (Ding et al., 2018; Kang et al., 2019).

Previously, we reported that the addition of sulfones to an electrolyte could increase the thermal stability, however electronically isolated deposits upon continued cycling were also observed (Merrill and Schaefer, 2018). Here, we report on the crystallinity and morphology of the magnesium deposits from electrolytes with and without the sulfone as a function of current density and substrate. We also probe the interfacial impedances throughout galvanostatic cycling and relate these findings to the morphologies observed. It was found that the sulfone based electrolytes result in high surface area deposition morphologies, such a hemispheres, compared to the electrolyte containing only THF; this is attributed to the influence of a boundary layer at the electrode that forms with the addition of the sulfone.

## Materials and Methods

### Materials and Electrolyte Solutions

Tetrahydrofuran (99.9%, THF), magnesium bis(hexamethyldisilazide) (97%, Mg(HMDS)_2_), di-n-butylsulfone (99%, BS), and magnesium chloride (99.99%, MgCl_2_) were purchased from Sigma Aldrich, and ethyl methyl sulfone (98%, EMS) was purchased from TCI. All solvents were distilled under nitrogen flow on a Schlenk line. Mg(HMDS)_2_ was recrystallized from anhydrous n-heptane (Sigma Aldrich) in a glovebox prior to use. All solvents and solvent mixtures were dried using molecular sieves for at least 48 h before making the electrolytes. Only new or acid washed glassware was used, and all glassware was dried in a 120°C convection oven. The electrolyte formulations were Mg(HMDS)_2_ – 4 MgCl_2_ (nominally 1.25 M Mg) in THF, 50 THF/50 BS (v/v), and 50 THF/50 EMS (v/v). Electrolytes were prepared as described in previous work (Liao et al., 2015; Merrill and Schaefer, 2018).

Magnesium (99.95%, Solution Materials, LLC), copper (McMaster Carr), and platinum (Alfa Aesar) were used as electrode materials for this study. Platinum was cleaned with concentrated nitric acid, then washed with MilliQ water, and dried in a 120°C convection. Copper was sonicated in isopropanol and then dried. Magnesium was scraped to remove its native oxide layer, then polished with 1,200, 2,000, and 3,000 grit sand paper, washed with anhydrous THF, then scraped again to further smooth the surface. Supplementary Figure 1 shows electron micrographs of the bulk magnesium surface before and after the final scraping step.

All electrolytes and electrochemical cells were prepared in an argon filled glovebox with <5 ppm oxygen and moisture. Coin cell measurements were completed outside of the glovebox.

### Electrochemical Measurements

All electrochemical measurements were taken using a PARSTAT MC1000 from Princeton Applied Research.

Galvanostatic electrodeposition was carried out in a 2 electrode solution cell. The working electrode was either magnesium, copper, or platinum and a magnesium strip was used as the counter/reference electrode. Currents of 1 and 0.5 mA/cm^2^ were applied until the total charge passed was 1 C/cm^2^ for microscopy characterization or 10 C/cm^2^ for X-ray diffraction (XRD) measurements.

Chronoamperometry measurements were completed using a 2 electrode solution cell. The working electrode was copper and the counter/reference electrode was magnesium. The cell was held at open circuit for 10 s followed by a potential step for 10 s. Overpotentials used include −250, −375, and −500 mV vs. Mg^0^/Mg^2+^.

Galvanostatic cycling and impedance were carried out in symmetric Mg/Mg CR2032 coin cells. The cells were assembled in an argon filled glovebox. A dried glass fiber separator was used to support the electrolyte. A current of −0.5 mA/cm^2^ was applied for 1,000 s followed by +0.5 mA/cm^2^ for 1,000 s. Impedance measurements were taken every five cycles, with an amplitude of 10 mV RMS and a frequency range of 10 kHz to 1 Hz.

### X-ray Diffraction (XRD)

XRD measurements were obtained using a Bruker D8 Advance Davinci with a SOL-XE energy dispersive x-ray detector. Measurements were taken in the range of 20 and 70°, with a step size of 0.004°, and a step time of 2.5 s. Only copper was used as the substrate for magnesium deposition for XRD measurements. Measurements were taken on a silicon crystal zero diffraction plate from MTI Corporation.

### Scanning Electron Microscopy (SEM)

SEM images were taken using an FEI Magellan 400 microscope with a voltage of 10 kV and a current of 50 pA at a working distance of 4.3 mm. Elemental analysis was completed using a Bruker energy dispersive x-ray spectrometer (EDS) at a working distance of 4.7 mm with an increased current to achieve adequate signal (>1,000 cps). Samples were washed with anhydrous THF then put under vacuum prior to characterization. A Pelco SEM pin stub vacuum desiccator was used to transfer samples from the lab to the microscope to minimize air exposure.

### X-ray Photoelectron Spectroscopy (XPS)

XPS measurements were taken using a PHI VersaProbe II. Point scans were taken with a 50 W power X-ray. Prior to measurements, samples were sputtered for 2 min with 2 kV Ar^+^ to clean the surface. Survey measurements were taken with a 187.85 eV pass energy over 7 scans. Each individual binding energy measurement was taken with a 23.5 eV pass energy for 12 scans. A 100 ms time step was used for all scans. Samples were washed with THF and dried under vacuum prior to analysis, then transferred to the instrument using the Pelco pin stub holder, however air exposure could not be completely avoided upon transfer into the machine's sample holder.

## Results and Discussion

### Morphologies of Magnesium Deposits

Magnesium was deposited from each electrolyte at different current densities onto copper, platinum, and magnesium. Previous reports of magnesium deposition showed that for a Grignard based electrolyte, increasing the current density will bring it into a mass transport limited regime. Once the system is mass transport limited, the magnesium will not be able to optimize its direction of growth (Viestfrid et al., 2005; Matsui, 2011). This can then result in the magnesium crystal growing in the perpendicular direction to the substrate or growing in the same direction as the crystal facets within the substrate. Low current results in operation in the charge transport limited regime. This allows the magnesium to nucleate on the substrate and grow in a thermodynamically controlled manner, often resulting in high surface area deposits. As shown in Supplementary Figure 2, the formation of spherical magnesium deposits can ultimately increase the effective local current density, leading to decomposition that is evident from the charging on the deposits in the SEM images. In this work, we focus on magnesium electrodeposition at moderate (0.5 mA/cm^2^) and high (1 mA/cm^2^) current densities.

**Figure 1:**
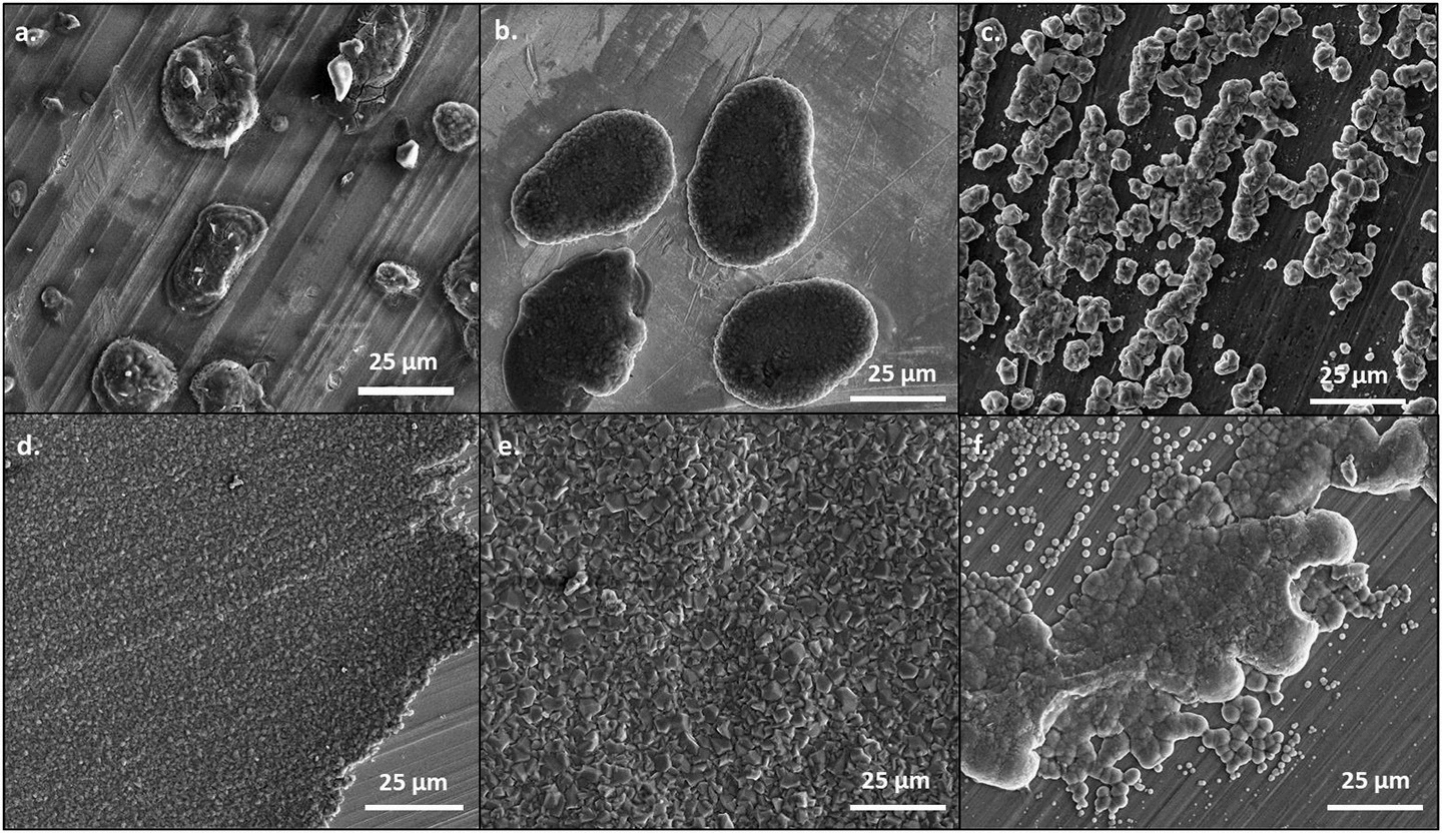
SEM images of magnesium plated at 0.5 mA/cm^2^. A total of 1 C of charge was passed for each sample. Magnesium metal was plated from the 50 BS/50 THF electrolyte on **(A)** copper, **(B)** platinum, and **(C)** magnesium; and from the THF electrolyte on **(D)** copper, **(E)** platinum, and **(F)** magnesium. All images are shown at a magnification of 1000x.

At 0.5 mA/cm^2^, there are significant differences in the morphology of the deposits from the 50 BS/50 THF electrolyte and THF electrolyte, as depicted in Figure 1. This is particularly evident on the copper and platinum substrates. The deposition from the 50 BS/50 THF electrolyte consists of deposits that are spaced apart, suggesting that the magnesium was able to nucleate at different points on the substrate, but the clusters of magnesium did not merge together. Areas of charging (apparent from areas of brightness on the SEM image), caused by non-conductive materials, are present around the edges of the spherical deposits. This non-metallic material could be produced due to electrolyte decomposition or a disruption in the electric field. The THF electrolyte exhibits very flat deposition composed of small, uniform crystals; an increased magnification of the SEM image is shown in Supplementary Figure 3. The magnesium clusters deposited from the THF electrolyte were able to merge after the initial nucleation. For both electrolytes, the deposition is more disperse on the bulk magnesium sheet metal, and appears to grow along the ridges of the bulk material; the deposits will typically form at imperfections on the metal that generated during the removal of the passivation layer by mechanical scraping. The deposit from the THF electrolyte has a greater degree of agglomeration compared to the deposit from the 50 BS/50 THF electrolyte.

**Figure 2:**
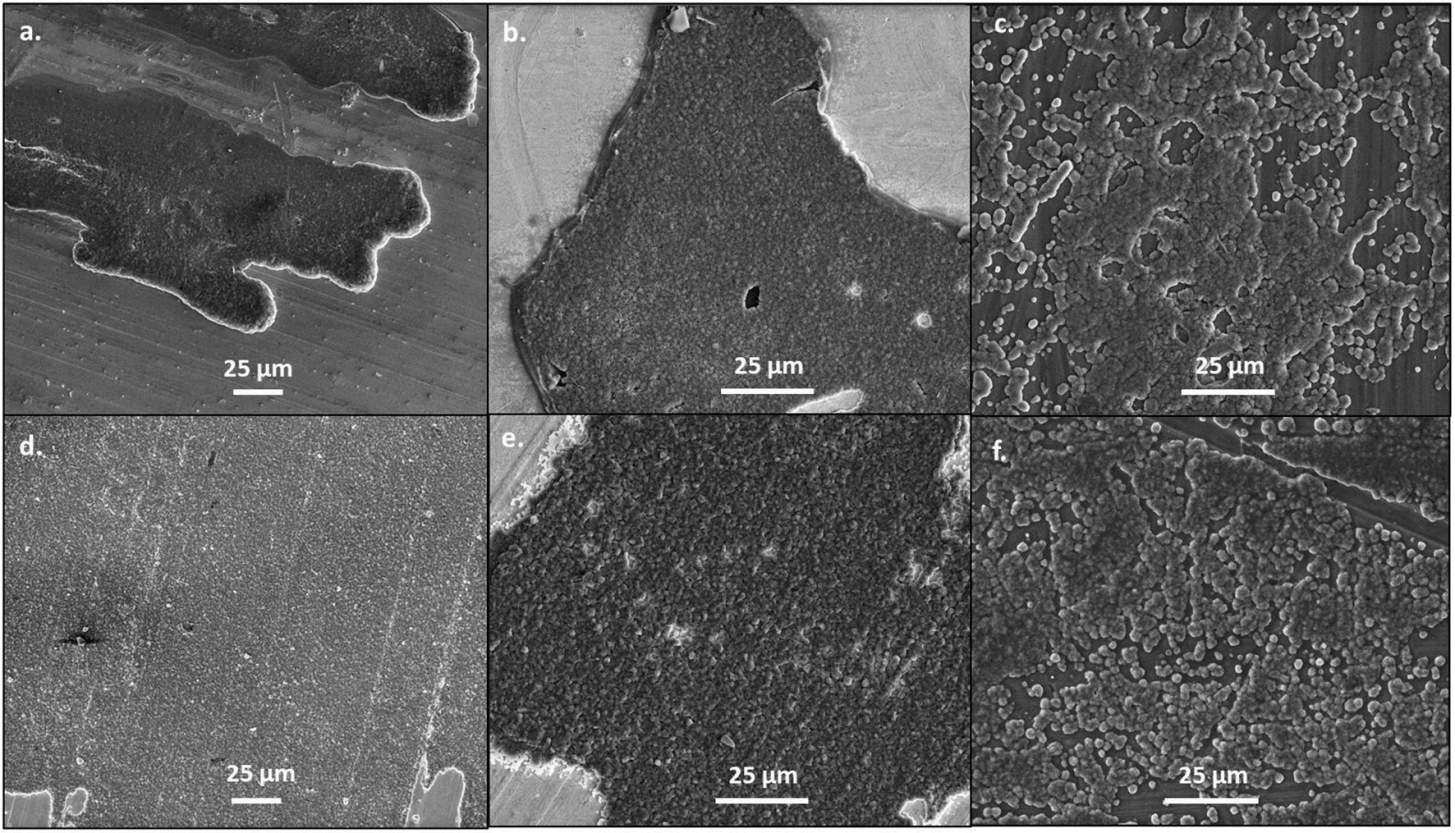
SEM images of magnesium plated at 1 mA/cm^2^. A total of 1 C of charge was passed for each sample. Magnesium metal was plated from the 50 BS/50 THF electrolyte on **(A)** copper, **(B)** platinum, and **(C)** magnesium; and from the THF electrolyte on **(D)** copper, **(E)** platinum, and **(F)** magnesium. All images are shown at a magnification of 1000x.

When the current density was increased to 1 mA/cm^2^, the deposits became flatter and more uniform, as shown in Figure 2. This is likely because the increased current density does not allow for the magnesium deposits to optimize the crystal orientation, like at lower currents. However, as shown in Supplementary Figure 4, the morphology of the magnesium deposit varies throughout the copper substrate, likely due to the influence of different crystal facets throughout the copper. The deposits from the 50 BS/50 THF electrolyte are more uniform and flat on the platinum substrate, compared to the copper, confirming that the substrate used will alter the interfacial chemistry. Again, the deposit from the THF electrolyte resulted in smooth deposition, in particular on copper. The deposition on the magnesium substrate resulted from the agglomeration of islands, however at the higher current each cluster is smaller, which is characteristic of the increased current density.

**Figure 3:**
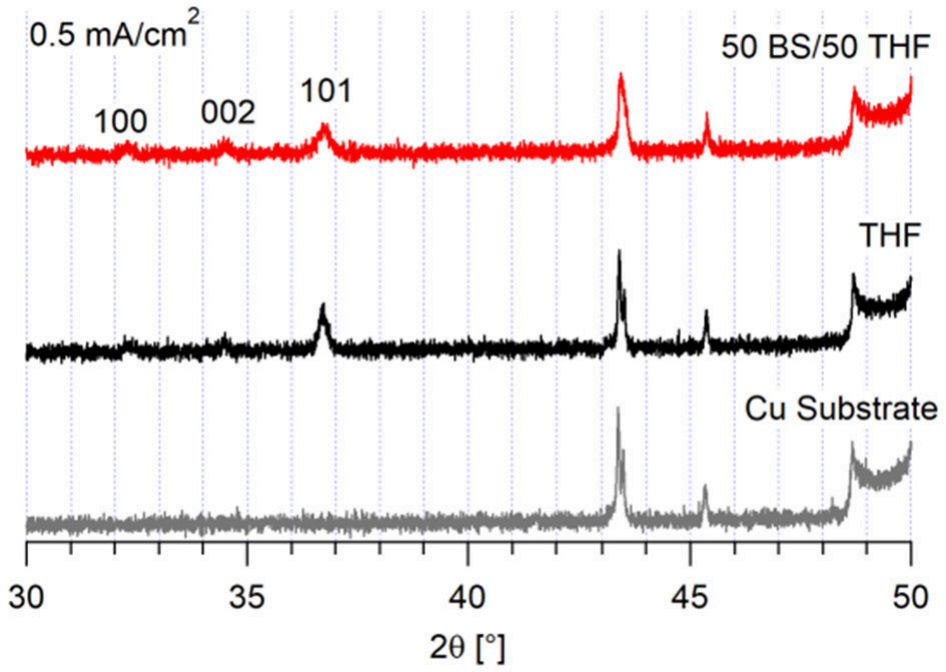
XRD of magnesium deposits on a copper substrate. 10 C of charge was passed at 0.5 mA/cm^2^.

As shown in Figure 3, the magnesium deposited at 0.5 mA/cm^2^ on copper preferentially grows along the (1 0 1) crystal plane for both electrolytes, which is the plane with the highest surface area fraction (Lautar et al., 2019). This observation suggests that the interactions between the electrolyte and the metal are more favorable. If the electrolyte-metal interface is thermodynamically preferred, then the deposit will grow such to maximize the surface area in contact with the electrolyte. The deposit from the 50 BS/50 THF electrolyte is less crystalline, evident from both decreased peak intensity and an increased full width half maximum, compared with the signal of the deposit from the THF electrolyte. The amorphous nature may be due to the high surface area nature of the deposits or due to a greater degree of decomposition.

**Figure 4:**
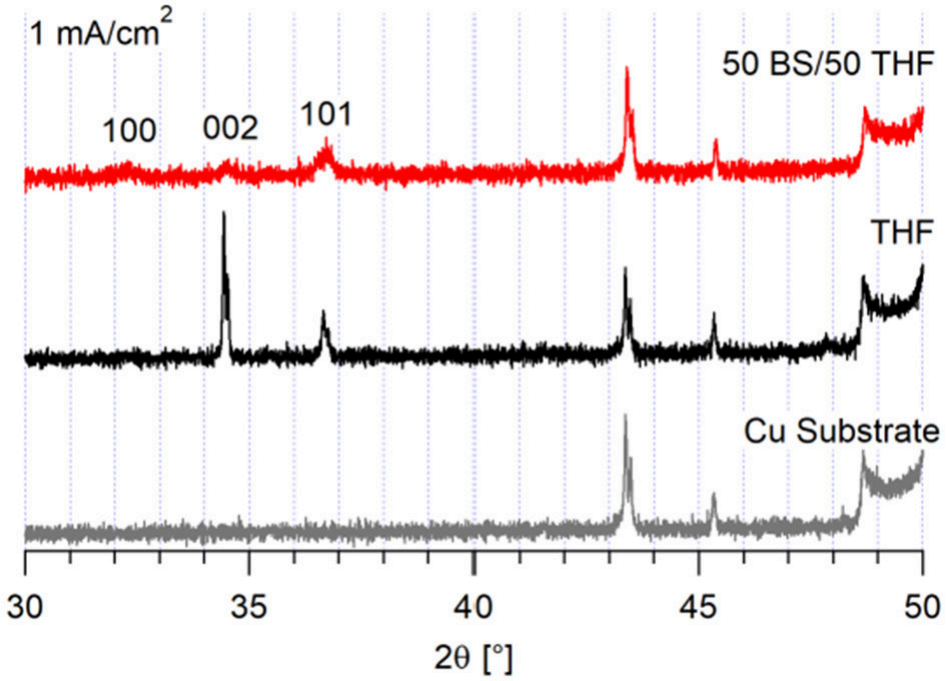
XRD of magnesium deposits on a copper substrate. 10 C of charge was passed at 1 mA/cm^2^.

The corresponding XRD of the deposits grown at 1 mA/cm^2^ show that different magnesium crystal orientations result from electrodeposition from each electrolyte, shown in Figure 4. The deposit from the THF electrolyte has increased crystallinity and a higher fraction of (0 0 2) orientation. This change in crystal orientation with current density suggests that the system with the THF electrolyte reached a transport limited regime at 1 mA/cm^2^. As shown in Supplementary Figure 5, the (0 0 2) plane is the primary plane in the copper substrate. The deposit from the 50 BS/50 THF electrolyte continues to deposit preferentially in the (1 0 1) plane, again with a lower degree of crystallinity. Because the 50 BS/50 THF electrolyte has a lower ionic conductivity (0.8 vs. 1.5 mS/cm at 30°C), it is unlikely that the THF electrolyte would enter a mass transport limited regime at a lower current than the 50 BS/50 THF electrolyte (Merrill and Schaefer, 2018). This suggests that the interfacial chemistry between the 50 BS/50 THF electrolyte and the substrate drives the resultant morphology, and that the interfacial chemistry with the THF electrolyte is different.

It is noted that the deposits from each electrolyte look different to the naked eye. The deposits from the THF electrolyte are a silvery-white color whereas the deposits from the 50 BS/50 THF electrolyte are black (see Supplementary Figure 6). As shown in our previous work, there is minimal decomposition therefore the resultant black color is likely related to the particle-like morphology (Merrill and Schaefer, 2018).

Chronoamperometry experiments were completed to further interrogate the deposition and nucleation processes for each electrolyte. The current response to a potential step is different for the two electrolytes, as shown in Supplementary Figure 7. The 50 BS/50 THF electrolyte begins with a large current, which immediately drops, followed by a slow increase and the beginnings of a plateau. This initial large current may be attributed to polarization of ions in the boundary layer at the electrode/electrolyte interface; this large current obscures the current due to the initial nucleation process in the 50 BS/50 THF electrolyte. In the transient for the THF electrolyte, a small local maximum is present, followed by similar behavior to the mixed solvent electrolyte. The local maximum is likely representative of the initial magnesium nucleation. Because the electron transfer reaction is hypothesized to be preceded by a chemical equilibrium step (Ta et al., 2018), it is likely that the chemical equilibrium step causes the initial decrease in current for each electrolyte. The Cottrell equation could not be fit to either electrolyte; therefore, the Scharifker and Hill models for instantaneous and progressive nucleation could not be directly applied to this system (Scharifker and Hills, 1983). Nevertheless, the local maxima in the THF electrolyte were used to plot a dimensionless graph of i^2^/i${}_{\text{m}}^{2}$ vs. t/t_m_ (Supplementary Figure 8), and suggest the formation of nucleation sites at short time scales. The complexity of the magnesium electrolytes are attributed to the changes from the ideal behavior, as previously derived to describe other systems (Scharifker and Hills, 1983); the magnesium electrolytes likely contain multiple electrochemically active complex cations with varying diffusivities. Additionally, the magnesium electrolytes facilitate boundary layer formation at the electrode/electrolyte interface when the system is at rest, as described below.

### Impedance and Cycling

Symmetric magnesium coin cells were cycled 100 times at 0.5 mA/cm^2^, starting with a negative current. Impedance measurements were taken every 5 cycles after the positive current. This was done to learn about the interfacial impedances throughout cycling and to determine if either passivation, or soft short circuiting, would be observed throughout extended cycling. Soft short circuiting is a phenomenon where the magnesium deposit is partially passivated and slowly grows through the separator, eventually making contact with the counter electrode, therefore short circuiting the cell. This has been observed with Mg(TFSI)_2_ electrolytes, which are unstable in the presence of magnesium metal (Ding et al., 2018; Kang et al., 2019).

**Figure 5:**
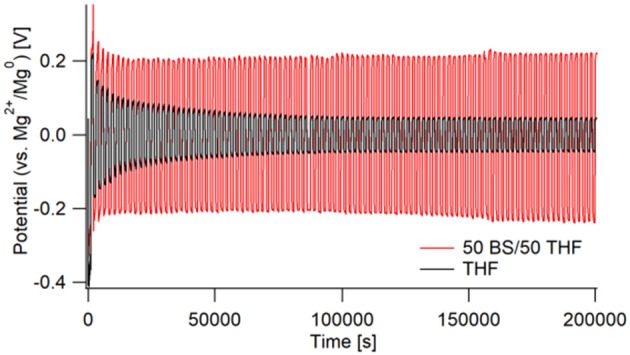
Galvanostatic cycling of Mg/Mg cells in 50 BS/50 THF (red) and THF (black). Cells were cycled at a current of 0.5 mA/cm^2^ for 1,000 s, starting with a negative current, then switching to a positive. This was repeated for 100 cycles with impedance measurements taken every 5 cycles.

As shown in Figure 5, there are very large differences in resistance for each electrolyte during galvanostatic cycling. The THF electrolyte initially starts with a high overpotential of ~0.4 V vs. Mg, which then decreases throughout cycling. The 50 BS/50 THF electrolyte shows a similar decrease in overpotential during the first few cycles, however a much greater overpotential is maintained throughout cycling (about 200 vs. 75 mV). This is in part due to the greater bulk resistance caused by the lower conductivity of the 50 BS/50 THF electrolyte. Therefore, to maintain the same current, a higher overpotential is required. Over extended cycling, there is a gradual increase in overpotential for the case with the 50 BS/50 THF electrolyte; indicating an increase in resistance. The cycling behavior of a wet THF electrolyte (40–60 ppm moisture) was similar to that of an electrolyte made with dried THF. This confirms that the large over potentials present in the chronopotentiogram are unique to the sulfone electrolyte, and not related to the existence of residual water or passivation of magnesium due to residual water.

**Figure 6:**
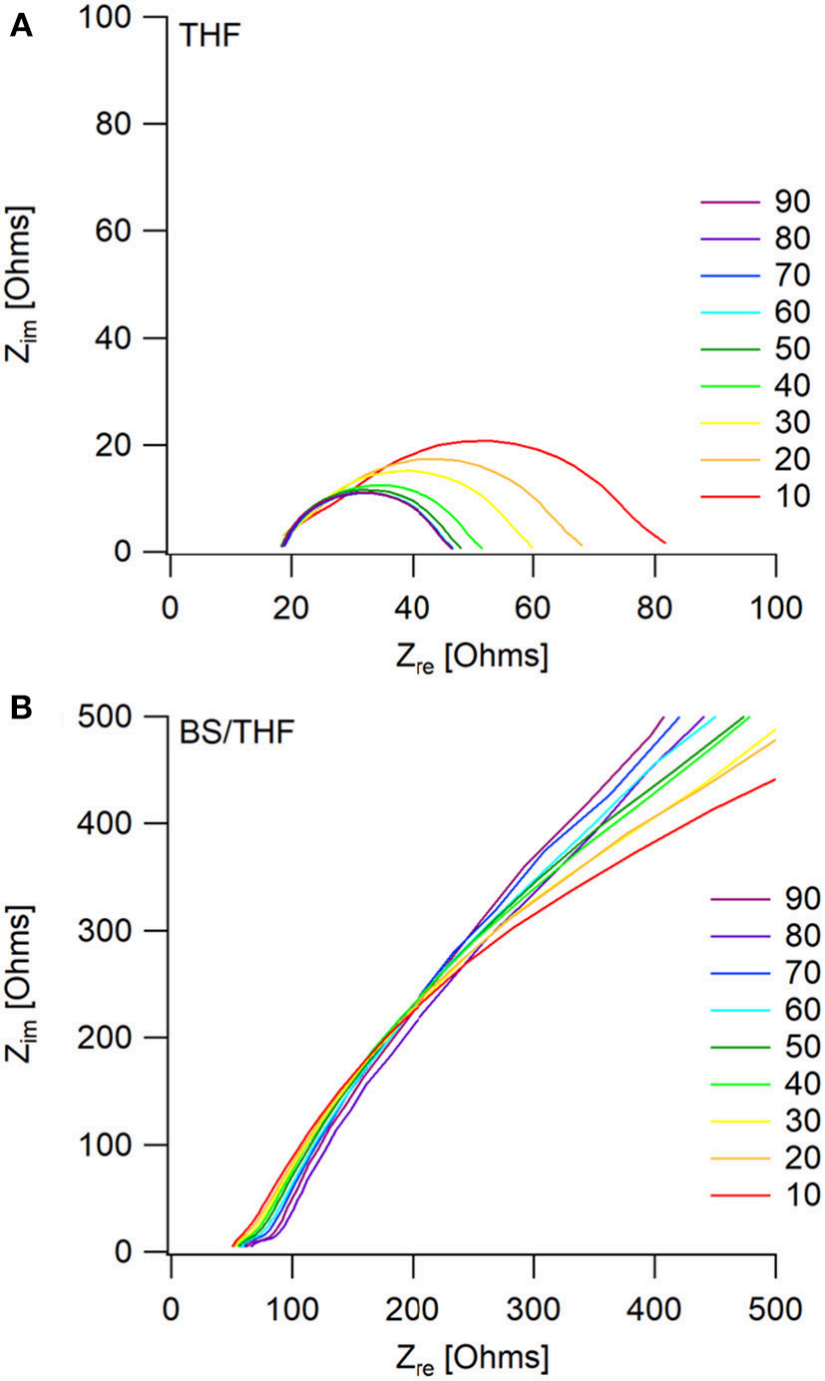
Impedance measurements taken every 5th cycle (only multiples of 10 are shown for visual clarity) after the positive current step, during galvanostatic cycling. Mg/Mg cells were cycled in **(A)** the THF electrolyte and **(B)** the 50 BS/50 THF electrolyte.

The corresponding Nyquist plots give more information about the impedances within the cells. For the THF electrolyte, there is a decrease in interfacial impedance throughout cycling until the cells appear to reach a steady state (around cycle 60), shown in Figure 6A. This is represented by the initial high overpotential, which then decreases and plateaus throughout cycling. The bulk impedance stays fairly constant throughout cycling. The decreasing interfacial impedance in the first 60 cycles is possibly due to a combination of two phenomena: one being the removal of trace water in the electrolyte, and the other due to a native surface layer that forms on the electrodes as previously described by the Aurbach group (Lu et al., 1999; Doron Aurbach et al., 2003; Viestfrid et al., 2005; Attias et al., 2019). Supplementary Figure 9 shows that the interfacial impedances for the wet THF and the dry THF electrolytes are comparable.

The similar decrease in overpotential during the first couple cycles of the 50 BS/50 THF electrolyte is likely due to the same phenomena as described with the THF electrolyte, however, a decrease in interfacial impedance is not observed, as shown in Figure 6B. Instead, there is a very large interfacial impedance present throughout cycling. A similar impedance is observed in the pristine cells for both electrolytes (Supplementary Figure 10), however after the application of a current, the interfacial impedance greatly decreases in the THF electrolyte. This impedance is caused by the formation of a boundary layer at the electrodes; because the sulfone group is polarizable and the alkane chains are able to stack, it is plausible that this boundary layer is stabilized. This boundary layer could be the cause of the high surface area deposition morphology by altering the interface. Provided the interaction between the metal and the boundary layer is more thermodynamically favorable, the deposit would grow to maximize its surface area to allow more of the metal to be in contact with the boundary layer.

The bulk resistance does increase with continued cycling, likely due to solvent loss via decomposition. As previously reported, the 50 BS/50 THF electrolyte is able to maintain Coulombic efficiencies around 90–95%, suggesting that there is a small amount of electrolyte decomposition (Merrill and Schaefer, 2018). Therefore, the gradual increase in bulk resistance and overpotential throughout cycling is related to the gradual buildup of a passivating surface layer. Over extended cycling the impedance continues to grow, suggesting that the cell will become fully passivated.

### Post Mortem Analysis

#### SEM/EDS

**Figure 7:**
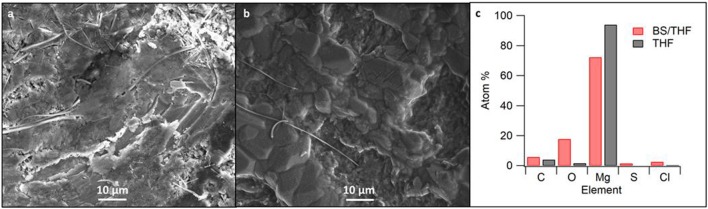
SEM images of cycled magnesium in **(A)** the 50 BS/50 THF electrolyte and **(B)** the THF electrolyte. SEM images were taken at 1000x magnification; **(C)** shows the corresponding elemental composition as detected by EDS measurements.

Upon post-mortem analysis of the cells, the cycling of the 50 BS/50 THF electrolyte caused the separator to turn black whereas the separator used with the THF electrolyte had minimal discoloration in it (Supplementary Figure 11). This suggests decomposition of the 50 BS/50 THF electrolyte and/or the growth of the magnesium through the separator. The electrode used with the THF electrolyte was silvery-white whereas the electrode used with the 50 BS/50 THF electrolyte remained gray with large amounts of the glass fiber separator sticking to it, evidence that the magnesium began to grow through the separator. The SEM images in Figures 7A,B show the magnesium electrodes after a final stripping step. The electrode cycled with the 50 BS/50 THF electrolyte had areas of charging indicating partial decomposition. The electrode cycled with the THF electrolyte was more compact with the appearance of a higher degree of crystallinity. Because the resultant deposits from the 50 BS/50 THF electrolyte prefer a particle-like morphology, it is likely that the deposits are able to grow through the separator. Furthermore, as discussed earlier, this type of deposition can result in high local current densities which can lead to solvent decomposition.

Elemental analysis of the SEM-imaged surfaces are presented in Figure 7C. Increased proportions of elements other than magnesium are present in the deposit from the sulfone-ether electrolyte. Given that the Coulombic efficiency is <100%, the non-magnesium species are likely due to a decomposition product, such as solvent decomposition during deposition. This is further discussed with the XPS data. Alternatively, it is possible that the mixed solvent electrolyte contains a higher degree of impurities; although the butyl sulfone was distilled, its purity has not been determined.

#### XPS of Cycled Electrodes

**Figure 8:**
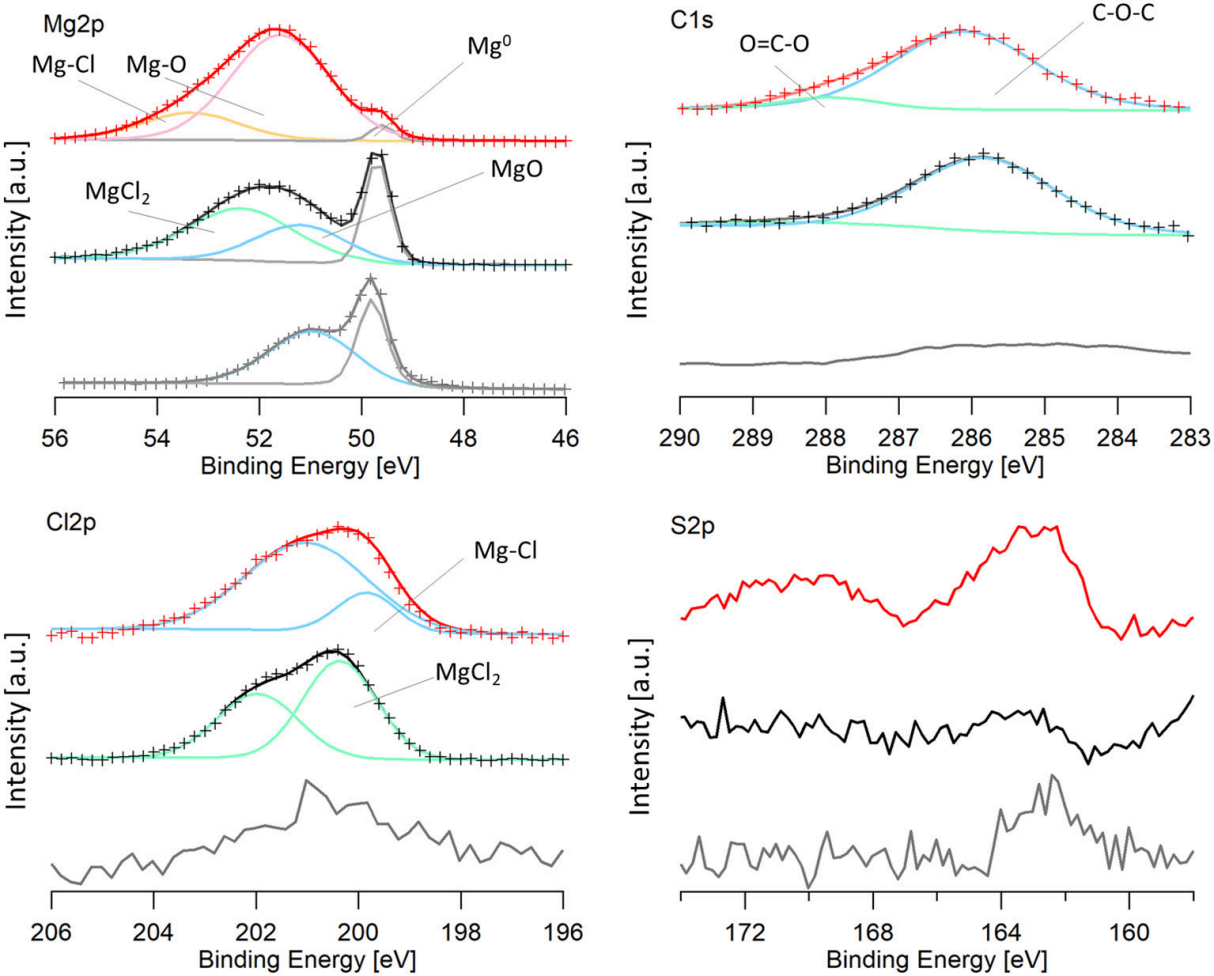
XPS spectra of an Mg electrode cycled in the 50 BS/50 THF electrolyte (red), an Mg electrode cycled in the THF electrolyte (black), and a pristine magnesium electrode (gray). For data that is fit: the experimental data is shown by the crosses and the line with the same color is the calculated fit. Peaks are shown by blue, green, pink, orange, and gray lines and are labeled on the image. Mg-Cl and Mg-O labels on the graph refer to a magnesium-chloride species (such as RMgCl or adsorbed MgCl) and a magnesium-oxygen species (such as Mg(OR)_2_ or Mg(OH)_2_) and are a different from MgCl_2_ and MgO.

Magnesium electrodes were characterized via XPS after the last dissolution step in the 100 cycles. The spectra from a pristine magnesium metal foil was included for a baseline comparison. Because oxidation was unable to be avoided upon transfer, as described in the experimental section, samples were sputtered for 2 min with Ar^+^. The spectra were calibrated to the lowest energy magnesium peak, assumed to be Mg^0^ (49.6 eV), because of the low carbon signal. The Mg2p, Cl2p, S2p, and C1s regions are shown in Figure 8, for the cycled electrodes.

It is immediately evident that both cycled electrodes have a higher degree of oxidation compared to the pristine magnesium. However, the sample cycled in the 50 BS/50 THF electrolyte has a larger amount of decomposition products present, evident from the smaller metallic magnesium peak. Some oxidation may be attributed to transfer, considering the pristine magnesium metal is partly oxidized. The electrode cycled in the THF electrolyte has an oxidation peak with the same binding energy as the oxidation of the pristine electrode, shown in blue, and it is likely due to the formation of magnesium oxide. This peak is shifted to a slightly higher binding energy for the electrode cycled in the 50 BS/50 THF electrolyte. This is likely due to an Mg-O bond as well, but is attributed to a different species (ex. Mg(OR)_2_ or Mg(OH)_2_), resultant from decomposition.

Both of the cycled electrodes show a higher binding energy peak in the magnesium XPS spectra that is due to an Mg-Cl bond, also identified in the Cl2p region. The active species is a magnesium chloride cation and an adsorption step precedes the electron transfer, which can cause the Mg-Cl peak to be present (Doron Aurbach et al., 2003; Viestfrid et al., 2005; Attias et al., 2019). For the magnesium electrode cycled in the THF only electrolyte, the Cl2p region contains MgCl_2_, evident from its binding energy around 200 eV (Magni and Somorjai, 1996). The residual MgCl_2_ on the cycled electrode is due to its low solubility in THF; similar reports have been shown for MgBr_2_ (Wetzel et al., 2015). The Cl2p peak for the electrode cycled in the mixed solvent electrolyte was shifted slightly lower (199.6 eV) (Moulder et al., 1992). This binding energy is evident of a metal-chloride (ex. RMgCl, or adsorbed MgCl), suggesting the formation of a different Mg-Cl species. This species may be resultant from the adsorption step as discussed above or a decomposition product. The Cl2p spectra for both electrodes show the presence of a secondary peak, which is due to the spin orbit splitting (Moulder et al., 1992).

The carbon spectra for magnesium electrodes cycled in each electrolyte features a major peak at 286 eV and a smaller peak at 288 eV which is characteristic of C-O-C and O-C=O, respectively. Because the XPS is carried out under high vacuum, it is unlikely that the major peak at 286 eV is due to THF solvent, but rather THF decomposition products, such as poly(ether). The O-C=O peak is also attributed to the decomposition of THF. Although THF-based electrolytes are reported to have high reversibility, THF has been shown to decompose on magnesium electrodes upon cycling in Grignard based electrolytes (Wetzel et al., 2015).

Unique to the 50 BS/50 THF electrolyte is the presence of sulfur compounds. In the S2p region, there is a small peak at 162 eV present in the 50 BS/50 THF sample. Because the intensities of these peaks are low, the peaks could not be properly fit, and the presence of a second sulfur species could not be verified. The peak at 162 eV is representative of a metal-sulfide bond, indicating that the sulfone, or a trace sulfur-containing impurity present in the electrolyte, reacted with magnesium to form an Mg-S bond.

### Other Sulfones

**Figure 9:**
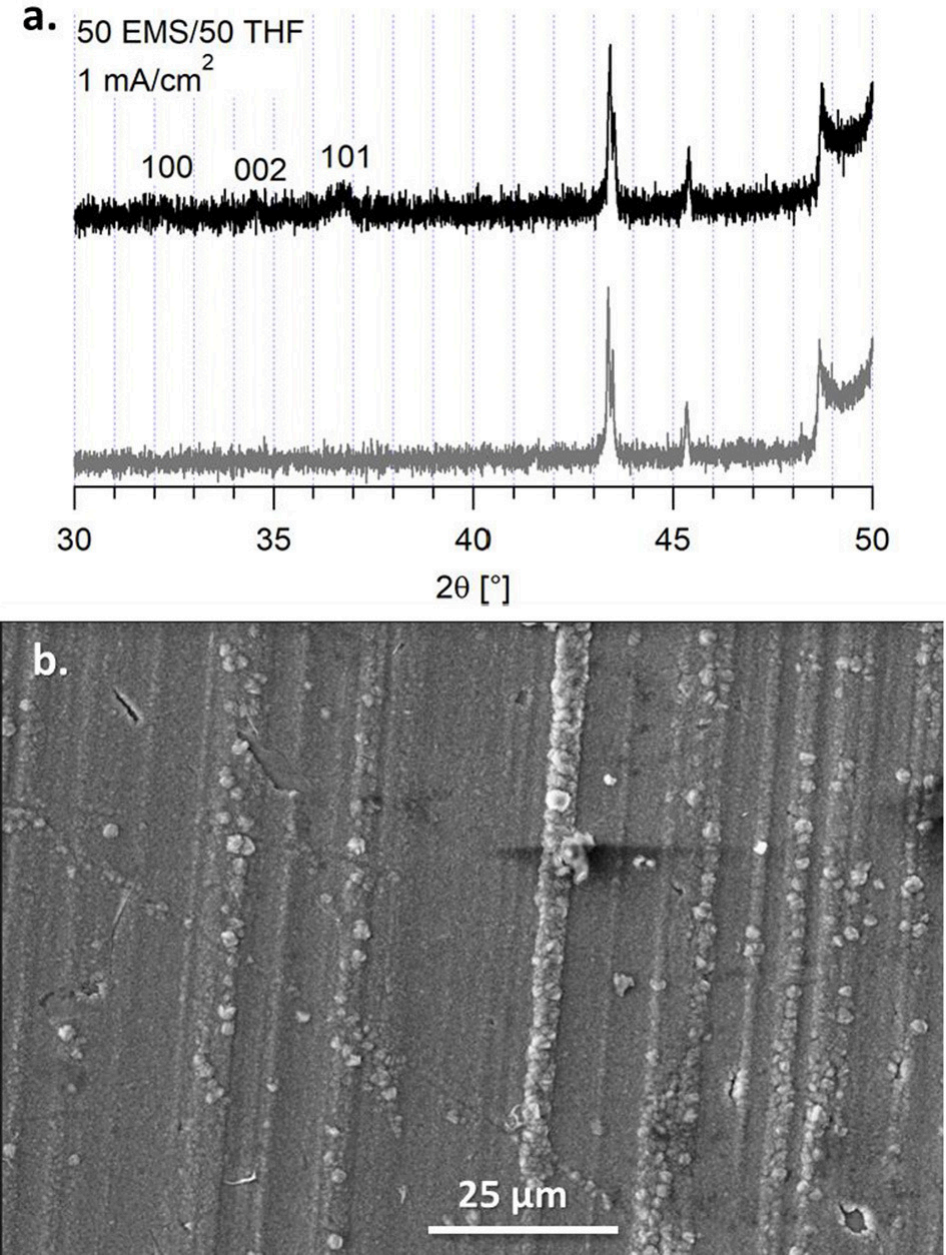
**(A)** XRD of the deposit from the 50 EMS/50 THF electrolyte on a copper substrate (black) and the copper substrate (gray). **(B)** SEM image of the deposit from the 50 EMS/50 THF electrolyte on a copper substrate at a current density of 1 mA/cm^2^, taken at a magnification of 1000x.

An alternative to butyl sulfone was studied to determine if the boundary layer effect observed in the EIS and the morphology of the magnesium deposits was due to incorporating a higher dielectric media or due to the stacking nature of the alkane chains. We previously compared the Mg(HMDS)_2_ – 4 MgCl_2_ electrolyte in 50 BS/50 THF to the electrolyte in sulfolane (SL)/THF (50/50, v/v), but this electrolyte was unable to support reversible magnesium deposition (Merrill and Schaefer, 2018). However, the electrolyte in a 50 EMS/50 THF mixture was able to support quasi-reversible magnesium electrodeposition. This electrolyte has a lower Coulombic efficiency, compared to the 50 BS/50 THF electrolyte, around 80% from cyclic voltammetry measurements (Supplementary Figure 12). Despite the flat, orderly deposition observed in the SEM image, Figure 9, the XRD still shows that the deposits are primarily amorphous. Because of the lower efficiency, decomposition may be causing the amorphous behavior of the magnesium deposit upon the extended potential hold. However, like with the 50 BS/50 THF electrolyte, the presence of a boundary layer can influence the deposition morphology.

**Figure 10:**
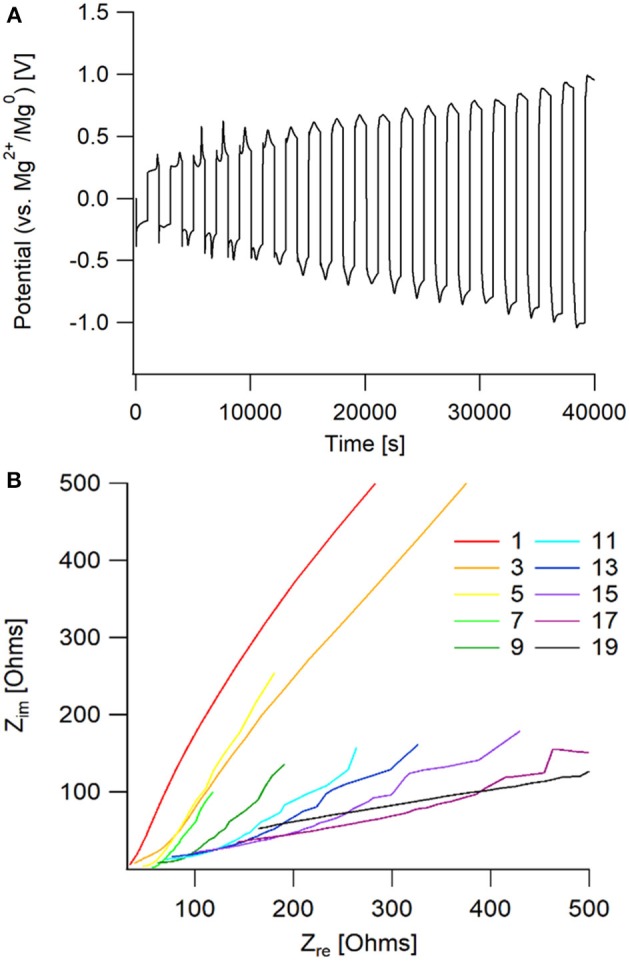
**(A)** Potential vs. time graph of galvanostatic cycling of an Mg/Mg cell with the 50 EMS/50 THF electrolyte. **(B)** Nyquist plot of the impedance taken every other cycle for the galvanostatic cycling. Impedance measurements were taken after completion of the full cycle, following the positive current step.

This particular electrolyte was not able to maintain extended cycling in a symmetric cell, shown in Figure 10. The passivation layer that grew on the magnesium metal caused an increasing resistance, that lead to the system reaching the voltage threshold of ± 1.5 V vs. Mg^2+^/Mg^0^. From the Nyquist plot, it is observed that there is a large increase in bulk impedance within the first 20 cycles, indicating that there was significant solvent decomposition. However, prior to the bulk resistance increase, the same impedance behavior to the 50 BS/50 THF electrolyte is observed in the early cycles. This confirms that the sulfone group is responsible for boundary layer formation at the electrode surface.

## Conclusions

It was determined that both substrate and current density have an influence on the microscopic properties of magnesium electrodeposits. More importantly, it was shown that the use of a sulfone/THF solvent mixture dramatically affects the deposition quality by changing the interfacial chemistry, leading to thermodynamically controlled deposition. It is hypothesized that the addition of sulfones to the electrolyte creates a boundary layer at the electrode-electrolyte interface due to the adsorption of the sulfone groups to the metal. It is likely that this boundary layer is what influences the formation of spherical deposits, due to the surface energies between the metal and the sulfone boundary layer.

The aforementioned particle-like deposition can be caused by the application of low current densities, prior to entering a mass transport limited regime. Increasing the current density can lessen the extent of spherical deposits, provided the interfacial chemistry, or thermodynamics, does not drive the deposition morphology—as is the case with the electrolytes containing sulfones. The high surface area deposits can change the effective current density, which results in areas of decomposition. Furthermore, upon extended cycling, non-uniform deposition into the pores of the separator is facilitated.

## Data Availability

All datasets generated for this study are included in the manuscript and/or the Supplementary Files.

## Author Contributions

JS conceived and guided the study. LM planned and conducted the experiments and data analysis. Both authors contributed to the writing of this paper.

### Conflict of Interest Statement

The authors declare that the research was conducted in the absence of any commercial or financial relationships that could be construed as a potential conflict of interest.
