# Supplementary material for: The Influence of Interfacial Chemistry on Magnesium Electrodeposition in Non-nucleophilic Electrolytes Using Sulfone-Ether Mixtures
Source: Front Chem. 2019 Apr 3;7:194. doi: 10.3389/fchem.2019.00194 (PMC6456701; doi:10.3389/fchem.2019.00194)
Supplement: Supplementary file 1 [file Data_Sheet_1.pdf]

*Supplementary Material*

# The influence of interfacial chemistry on magnesium electrodeposition in non-nucleophilic electrolytes using sulfone-ether mixtures

**Laura C. Merrill[1], Jennifer L. Schaefer[1*]**

[1]Department of Chemical and Biomolecular Engineering, University of Notre Dame, Notre Dame, IN, USA

*** Correspondence:**
Jennifer L. Schaefer
Jennifer.L.Schaefer.43@nd.edu

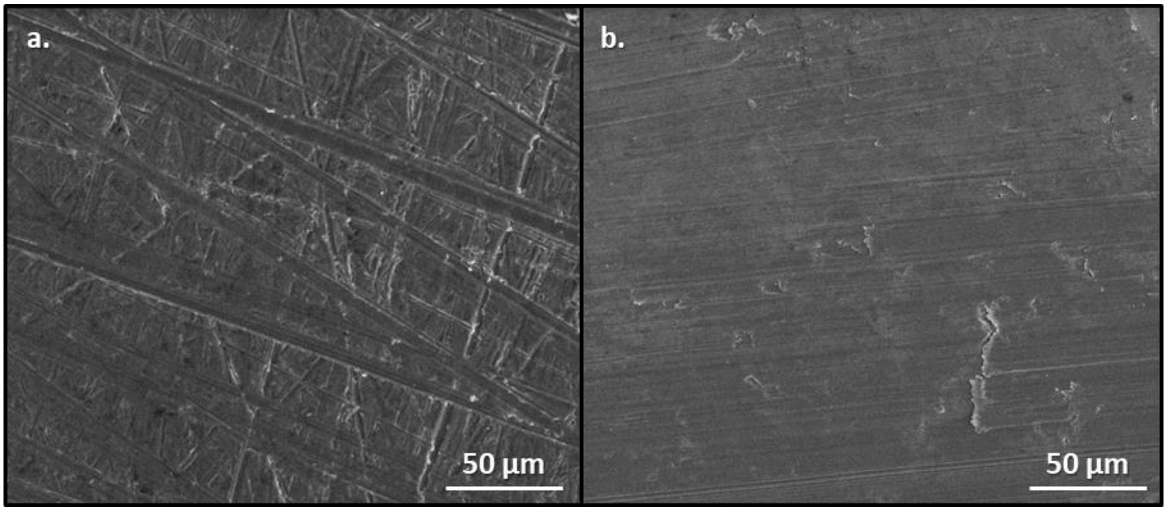


**Supplementary Figure 1:** SEM images of magnesium metal (a) after polishing with the 3000 grit sandpaper and (b) after scraping the magnesium following the polishing step. Images shown were taken at 500x magnification.

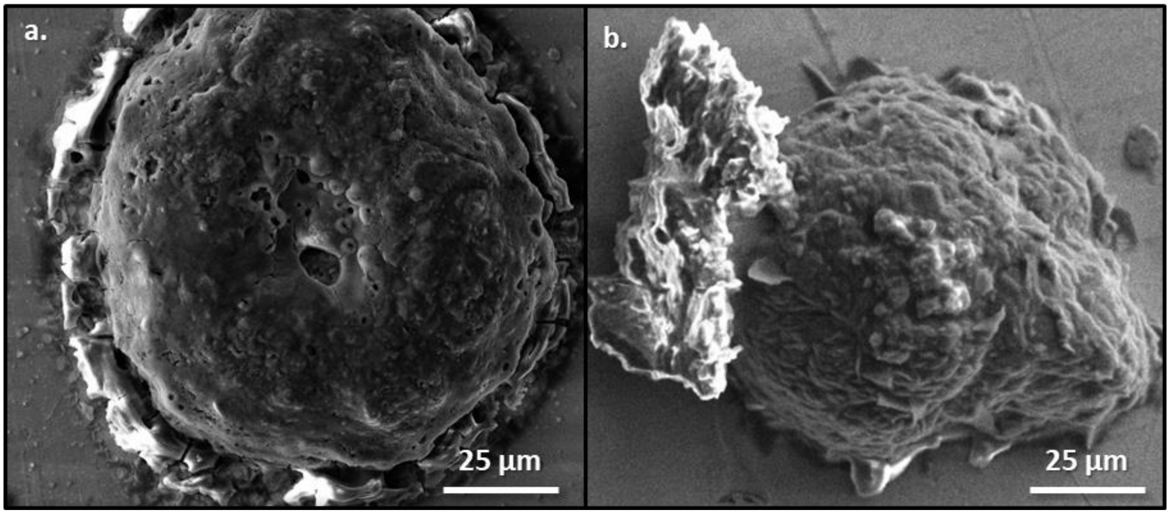


**Supplementary Figure 2:** SEM images of magnesium deposited at 0.1 $mA/cm^2$ on copper in (a) the THF electrolyte and (b) the 50 BS/50 THF electrolyte. The images shown are at 1000x magnification.

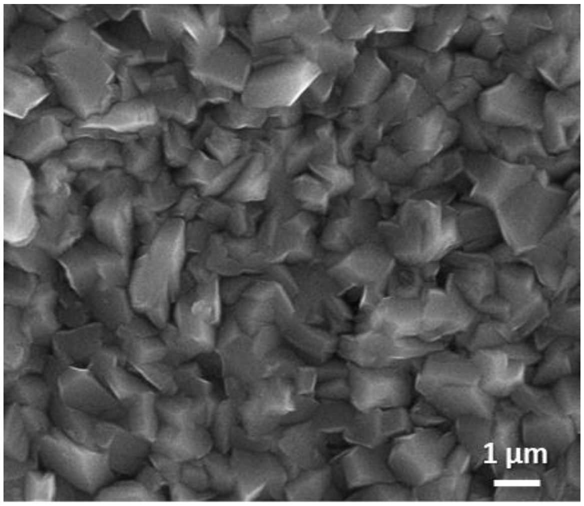


**Supplementary Figure 3:** SEM image of magnesium deposited from the THF electrolyte at 0.5 mA/cm$^2$ on copper at 10000x magnification.

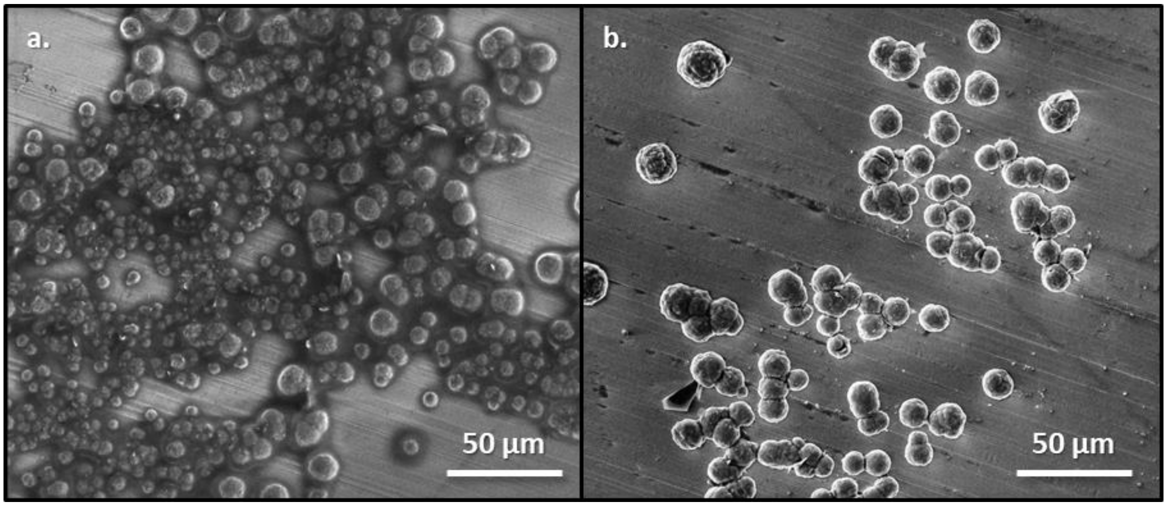


**Supplementary Figure 4:** SEM image of magnesium deposited on copper from the 50 BS/50 THF electrolyte at 1 mA/cm$^2$ at 1000x magnification. This is the same sample showed in Figure 2a.

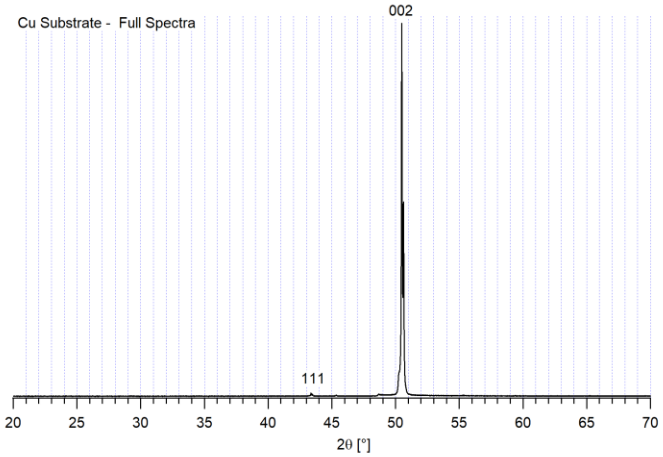


**Supplementary Figure 5:** XRD of the copper substrate.

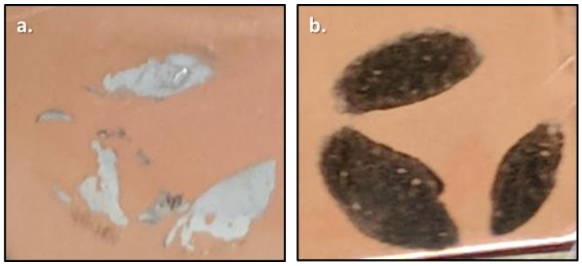


**Supplementary Figure 6:** Images of the deposit (a) from the THF electrolyte and (b) from the 50 BS/50 THF electrolyte

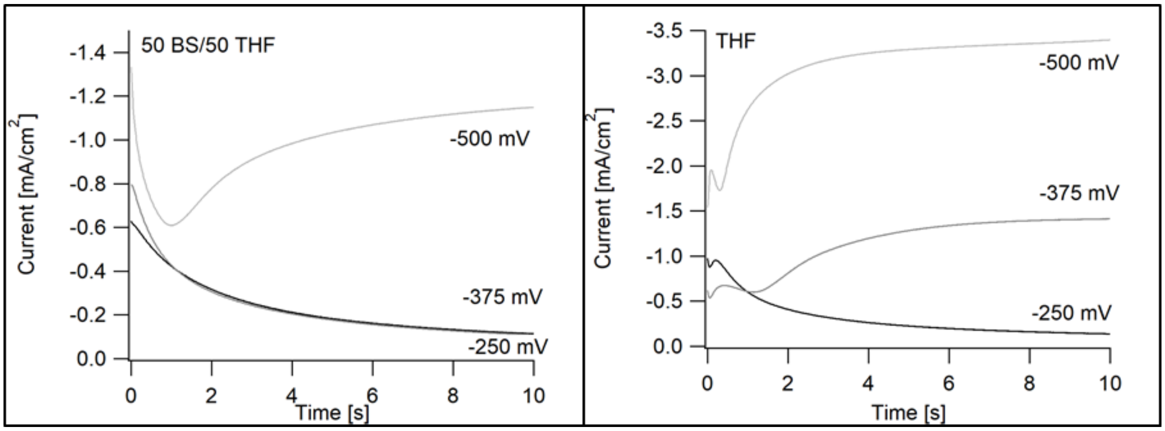


**Supplementary Figure 7:** Chronoamperometry of magnesium during electrodeposition onto a copper electrode for $Mg(HMDS)_2$ – 4 $MgCl_2$ in THF and in 50 BS/50 THF at -250, -375, and -500 mV vs. Mg.

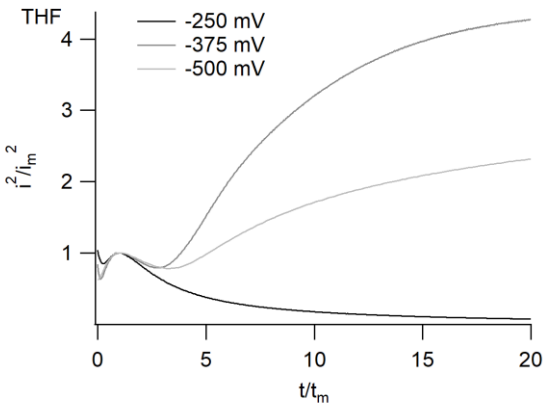


**Supplementary Figure 8:** Dimensionless graph of $i^2/i_m^2$ vs $t/t_m$ for the electrolyte in THF at -250, -375, and -500 mV vs. Mg. The maximum current, $i_m$, and maximum time, $t_m$, were chosen from the local maxima present in Figure S5.

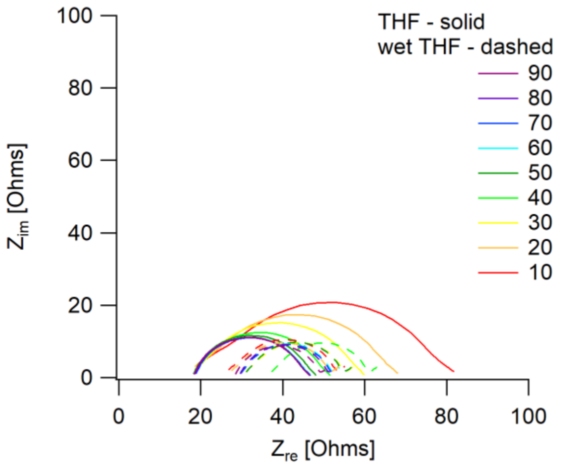


**Supplementary Figure 9:** Nyquist plots from every 10th cycle after a positive current. The electrolyte made with dried THF is shown with the solid lines and the electrolyte made with wet THF is shown by the dashed lines.

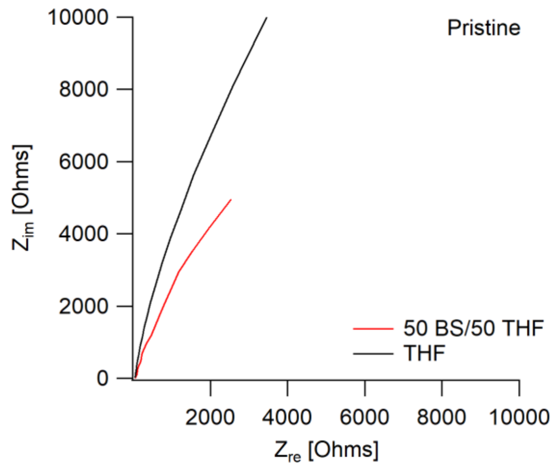


**Supplementary Figure 10:** Nyquist plot showing the impedance of the pristine Mg/Mg cells.

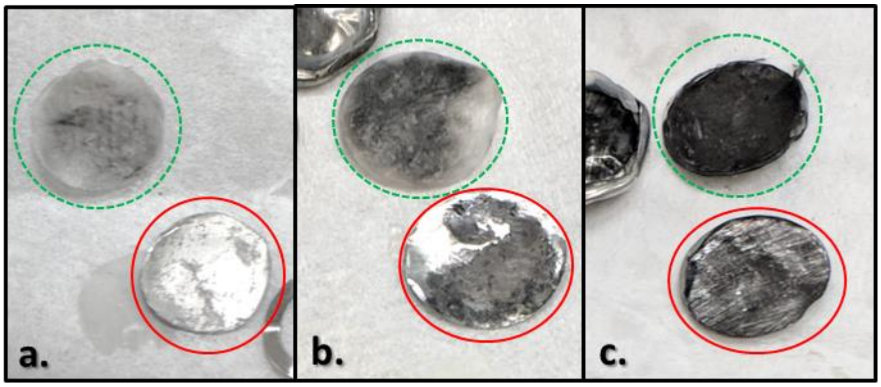


**Supplementary Figure 11:** Camera images of the cells post cycling in (a) the THF electrolyte, (b) the 50 BS/50 THF electrolyte, and (c) the 50 EMS/50 THF electrolyte. The green dashed circle identifies the glass fiber separator and the red solid circle identifies the cycled magnesium electrode after a final stripping step.

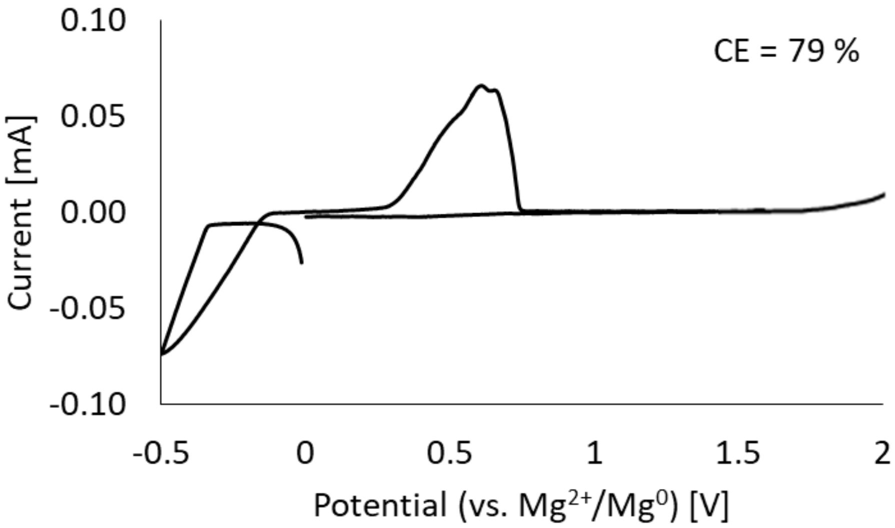


**Supplementary Figure 12:** Cyclic voltammogram of the 50 EMS/50 THF electrolyte. Copper was used as the working electrode and magnesium was used as the counter/reference electrode. Oxidative stability was approximately 1.9 V vs. $Mg^{2+}/Mg^{0}$.
